# Overexpression of long non‐coding RNA NORAD promotes invasion and migration in malignant melanoma via regulating the MIR‐205‐EGLN2 pathway

**DOI:** 10.1002/cam4.2046

**Published:** 2019-03-07

**Authors:** Yong Chen, Ke Cao, Jingjing Li, Aijun Wang, Lichun Sun, Jintian Tang, Wei Xiong, Xiao Zhou, Xiang Chen, Jianda Zhou, Yan Liu

**Affiliations:** ^1^ Department of Plastic Surgery of Third Xiangya Hospital Changsha China; ^2^ Surgical Department Emergency Department The First Hospital of Changsha Changsha China; ^3^ Department of Oncology of Third Xiangya Hospital Changsha China; ^4^ Surgical Bioengineering Laboratory Department of Surgery UC Davis School of Medicine Sacramento California; ^5^ Medicine School of Tulane University Health Science Center New Orleans Louisiana; ^6^ Institute of Medical Physics and Engineering Department of Engineering Physics Tsinghua University Beijing China; ^7^ Cancer Research Institute Key Laboratory of Carcinogenesis of Ministry of Health Changsha China; ^8^ Department of Head and Neck Surgery Department of Oncology Plastic Surgery Hunan Province Cancer Hospital Changsha China; ^9^ Department of Dermatology of Xiangya Hospital Changsha China

**Keywords:** long non‐coding RNA, melanoma, microRNA, oncogene

## Abstract

Growing evidence suggests that long non‐coding RNAs NORAD and miR‐205 play a significant role in regulating cancer progression and metastasis. In this study, high expression of NORAD was firstly observed in melanoma tissues and human malignant melanoma cell lines, our aim was to study the interaction of them in the process of invasion and migration of malignant melanoma cells. NORAD, miR‐205, and EGLN2 mRNA level in MM cells was detected by qRT‐PCR. In situ hybridization (ISH) was performed to detect NORAD expression in MM tissues specimens. Effects of NORAD and miR‐205 on Prolyl hydroxylase 2 (EGLN2) expression was explored by western blot in MM cells line. Dual‐luciferase reporter assay was performed to verify the interaction relationship between NORAD and miR‐205, as well as, miR‐205 and EGLN2. Transwell assay was conducted to explore the effects of NORAD and miR‐205 in vitro. Xenografts in nude mice experiment were used to confirm the role of NORAD and miR‐205 in vivo. In vitro, NORAD knockdown significantly inhibited migration and invasion of malignant melanoma cells and elevated the expression of miR‐205, there was an interaction between miR‐205 and NORAD in the RNA‐induced silencing complex. Upregulation of miR‐205 induced significant inhibition of migratory and invasive ability compared with the scrambled control. However, downregulating NORAD largely reversed this effect. Furthermore, the regulatory effects of miR‐205 on EGLN2 levels and the induction of endoplasmic reticulum stress were reversed by NORAD. In vivo, deletion of miR‐205 induced tumor growth in nude mice. NORAD may play critical roles in tumorigenesis and progression of malignant melanoma by regulating of the miR‐205‐EGLN2 pathway, and may serve as a new therapeutic target.

## 1 INTRODUCTION

Malignant melanoma (MM) is an aggressive skin cancer that accounts for 78% of skin cancer‐associated deaths.^1^, ^2^ The incidence of MM is increased by approximately 3% annually all over the world.^3^ Although significant advances have been obtained in diagnosis and treatments of MM, the prognosis of MM remains too poor and it still causes nearly 50 000 deaths per year worldwide.^4^ Therefore a better understanding of the underlying molecular mechanism of melanoma is important to develop adjuvant or alternative therapies and improve treatment efficacy.

Long non‐coding RNAs (lncRNAs) are non‐protein‐coding RNAs comprised of over 200 nucleotides. They are important for various cellular processes, such as cell growth, cycle progression, invasion, and metastasis.^5^, ^6^ In addition, growing evidence suggests that some lncRNAs can promote or suppress tumor growth in MM.^7^ For example, lncRNA MALAT1 (metastasis‐related lung adenocarcinoma transcript 1) is upregulated in uveal melanoma tissues and cells, and silencing of MALAT1 suppresses uveal melanoma cell proliferation, colony information, invasion, and migration.^8^ The long non‐coding RNA growth arrest‐specific transcript 5 (GAS5) has an anticancer function in melanoma via the mediation of gelatinase A and B activities.^9^

The non‐coding RNA activated by DNA damage (NORAD), also known as LINC00657, is located at 20q11.23. NORAD is highly conserved, it maintains genomic stability by repressing the stability and translation of mRNAs sequestering the PUMILIO protein.^10^ Previously, we reported that NORAD can act as a promoter of cellular stress responses in melanocytes.^11^ Moreover, NORAD knockdown suppresses the growth and proliferation of breast cancer cells, suggesting that NORAD may play an oncogenic role in breast cancer.^12^ However, the functions of NORAD in MM are still unknown.

Presently, we demonstrated the elevated expression of NORAD in MM tissues and cells. We also examined the effects and underlying mechanisms of NORAD expression in melanoma cells. Our findings clarify the significance of lncRNAs in MM and may provide an additional insight into the role of NORAD in the progression of MM.

## 2 MATERIALS AND METHODS

### 2.1 Cell transfection

Including paraffin‐embedded primary MM (62 cases), and normal skin tissues (20 cases) was purchased from Auragene Co. Ltd (cat No. TC0229; Changsha, China) for in situ *hybridization* confirmation of NORAD expression. Human melanocytes (HM) and four human MM cell lines (A375, WM451, SK‐MEL‐24, and WM35) purchased from the American Type Culture Collection (Manassas, VA) were cultured in RPMI‐1640 medium (Invitrogen, Carlsbad, CA) supplemented with 10% fetal bovine serum (Gibco, Carlsbad, CA) in a humidified atmosphere of 5% CO_2_ at 37°C. Ectopic miR‐205 expression was observed after transfection with either pre‐miR‐205 or anti‐miR‐205 (Genepharma, Shanghai, China) using Lipofectamine 3000 (Invitrogen). Knockdown of NORAD was performed using lentivirus (Lv) containing short hairpin (sh)RNA sequences of NORAD (GeneCopoecia, Guangzhou, China). Cells were cultured in either 6‐well clusters or 96‐well plates for 24 or 48 hours, and then used to experiments or RNA/protein extraction.

### 2.2 In situ hybridization (ISH)

The ISH was performed to detect NORAD expression in tissues specimens using digoxin‐labeled probe designed and synthesized by Sangon Biotech Co. Ltd. (Shanghai, China). The NORAD probe was TCTACTTCTGTCATACATTGGC) . The Enhanced Sensitive ISH Detection kit I (catalog no. MK1030; Wuhan Boster Biological Technology Ltd., Wuhan, China) was used to process the sample slices according to the manufacturer's protocol. The slides were then treated using 3, 3′‐diaminobenzidine (DAB; Fuzhou Maixin Biotech Co. Ltd., Fuzhou, China) for 5 minutes and counterstained with hematoxylin for 90s. At last, the slides were mounted and dried. Images of slides were captured with a model BX51 microscope (Olympus Corporation, Tokyo, Japan) at a magnification of 200× or 400×.

### 2.3 Quantitative real‐time reverse transcription PCR (qRT‐PCR)

Total RNA was extracted using Trizol reagent (Invitrogen). Mature miR‐205 expression was detected using a Hairpin‐it^™^ miRNA qRT‐PCR kit (Genepharma, Shanghai, China) in line with the manufacturer's instructions. Expression of RNU6B served as an endogenous control. NORAD expression was measured using the SYBR green qRT‐PCR assay (Takara Bio, Dalian, China). Expression of β‐actin was used as an endogenous control. The primers used were: NORAD, sense, CCTGGAAGGTGAGCGAAGT, anti‐sense, AGAGGGTGGTGGGCATTT; and β‐actin, sense, AGGGGCCGGACTCGTCATACT, anti‐sense, GGCGGCACCACCATGTACCCT. qRT‐PCR was performed at 95.0°C for 3 minutes, followed by 39 circles at 95.0°C for 10 seconds and 60°C for 30 seconds. Data were analyzed using the 2^−ΔΔCT^ method.

### 2.4 Transwell assay

Tumor cell invasion and migration capacities were assessed using Transwell inserts with 8 μm pores (Corning, NY). To assess invasion capacity, after transfection for 24 hours, cells (3.0 × 10^5^) cultured in serum‐free medium were added to the apical chamber that was pre‐coated with Matrigel matrix (BD, Franklin Lakes, NJ). FBS (10%, 500 μl) was dispensed in the matching basolateral chamber. After incubation for 48 hours, non‐invading cells that remained on the upper chamber surface of the transwell membrane were removed using a cotton swab. Invading cells that had traversed the membrane were fixed with methanol and stained with 0.1% crystal violet. Images were obtained and the cells were enumerated. The migration assay was performed the same way, except that 2 × 10^5^ cells were added into the chambers without a pre‐coated matrix gel. Six fields at 100× magnification were randomly selected and the cells were counted. Each experiment was conducted three times.

### 2.5 Western blot analysis

The expressions of EGLN2, glucose‐regulated protein 78 (GRP78), enhancer‐binding protein homologous protein (CHOP), eukaryotic elongation factor 2 kinase alpha (eIF2α), and phospho‐eIF2α (p‐eIF2α) in MM cell lines were determined by western blot analysis. The cells were lysed by RIPA buffer with 1 phenylethylsulfonyl fluoride. The proteins were separated using minigel SDS‐PAGE and transferred to a polyvinylidene fluoride membrane. After incubation with primary antibody (anti‐EGLN2, anti‐GRP78, anti‐CHOP, eIF2α, and anti‐p‐eIF2α; all from ImmunoWay Biotechnology Company, Plano, TX; anti‐β‐actin from Abcam, Cambridge, UK) at 4°C overnight, blots were incubated with horseradish peroxidase‐labeled secondary antibody (1:5000, Auragene, Changsha, China). Signals were developed by enhanced chemiluminescence (Millipore, Billerica, MA). β‐actin was used as the endogenous protein for normalization.

### 2.6 Dual‐luciferase reporter assay

NORAD (NCBI Reference Sequence: NR_027451.1) and the 3′UTR of EGLN2 (NCBI Reference Sequence: NM_053046.3) containing a putative miR‐205 binding site were synthesized by General Biosystem Co, Ltd. (Anhui, China). The qRT‐PCR products were subcloned and incorporated in a psiCHECK‐2 vector (Promega, Madison, WI). A psiCHECK‐2 construct with NORAD or 3′UTR EGLN2 with a mutant seed sequence was done to verify the constructs. The mutant sequence of NORAD and EGLN2 are as follows:

NORAD mut 5′…AGGAGTTGGAACCTTACTTCC…3′

EGLN2 mut 5′…CCGCTCGAGGCGTTACTTCCT..3′.

Luciferase activity was tested using a dual‐luciferase reporter assay system (Promega) normalized to *Renilla* luciferase activity.

### 2.7 RNA immunoprecipitation (RIP)

RIP experiments were conducted using a Magna RIP RNA‐Binding Protein IP Kit (Millipore) together with Ago2 antibody (Cell Signaling, Danvers, MA) as described by the manufacturers. Purified RNAs in the precipitates were used to determine NORAD and miR‐205 expression.

### 2.8 Xenografts in nude mice

All animal experiments complied with the NIH Animal Care Guidelines, and all experiments were approved by the Ethics Committee of the Faculty of Experimental Animals, Central South University, Changsha, China. Male BALB/c‐nu/nu (4‐6‐weeks of age raised in the animal laboratory of the Third Xiangya Hospital, Central South University) were kept in specific pathogen‐free conditions. Six nude mice were subcutaneously injected in the ventral trunk with 2 × 10^6^ cells in 200 μL Dulbecco's modified Eagle's medium. Tumor volume was calculated as V (mm^3^) = 0.5 × a × b^2^, where a is the maximum length to diameter and b is the maximum transverse diameter. Nude mice were sacrificed to assay the tumor volume and weight 35 days after tumor implantation.

### 2.9 Statistical analyses

SPSS 17.0 statistical software (SPSS Inc., Chicago, IL) was used. All measurement data are expressed as mean ± SD. The difference between the two groups was estimated by Student's *t*‐test, and difference among multiple groups was analyzed by one‐way ANOVA. A value of *P *<* *0.05 was considered statistically significant.

## 3 RESULTS

### 3.1 NORAD is upregulated in melanoma tissues and cell lines

**Figure 1:**
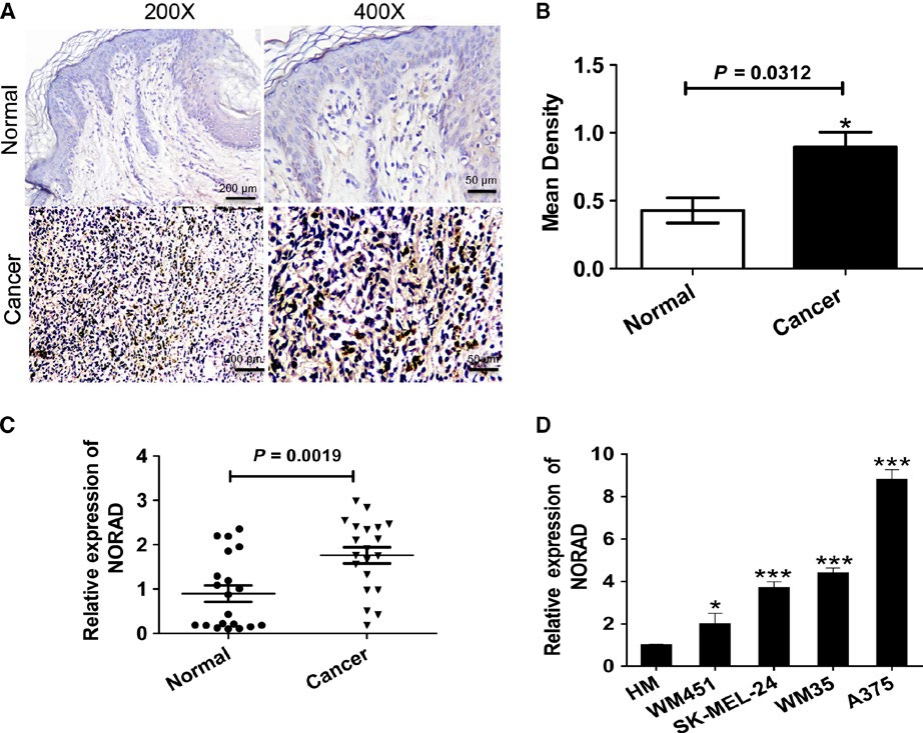
NORAD is frequently downregulated in MM tissues and cell lines. (A) ISH was performed to detect the expression of NORAD in MM tissues and normal skin tissues. (B) IPP6.0 software was used to analyzed the results of the ISH assay for NORAD expression. (C) The expression of NORAD in MM tissues was measured by qRT‐PCR. (D) qRT‐PCR was also used to detect the expression of NORAD in MM cell lines and normal human melanocytes (HM). *P <* *0.05, **P <* *0.01, ***P <* *0.001 vs normal control (NC)

ISH and qRT‐PCR assays were used to detect NORAD level in melanoma tissues and normal tissues. ISH revealed higher expression of NORAD in MM tissues (62 cases) compared to normal tissues (20 cases) (Figure 1A and B). qRT‐PCR confirmed NORAD upregulation in melanoma tissues compared to normal tissues (Figure 1C). The expression of NORAD in human melanocyte and MM human cell lines (A375, WM451, SK‐MEL‐24, and WM35) were also measured. An obviously upregulation of NORAD in the melanoma cell lines was observed comparing with the control human melanocytes (Figure 1D), which implicated the upregulation of NORAD in MM.

### 3.2 Downregulation of NORAD inhibits MM cell migration and invasion

**Figure 2:**
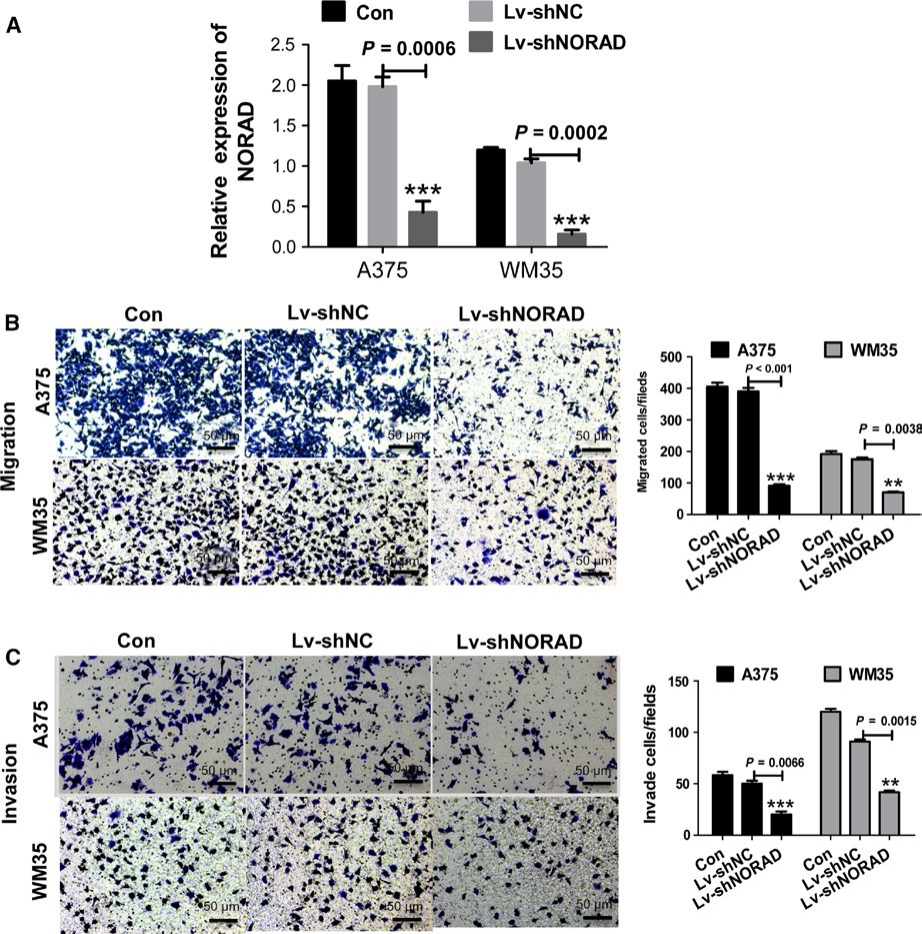
Downregulation of NORAD inhibits the migration and invasion of malignant melanoma cells. (A) qPCR was performed to detect the expression of NORAD in A357 and WM35 cells after transfection with Lv‐shNORAD. Cells treated with Lv‐shNC were used as control. (B) NORAD knockdown inhibited the migration of A357 and WM35 cells. (C) NORAD knockdown inhibited the invasion of A357 and WM35 cells. **P <* *0.01, ***P <* *0.001 vs normal control (NC) group. Images were obtained at 100× magnification

To assess whether NORAD affects the malignant behavior of A375 and WM35 cells or not, NORAD knockdown cells were constructed (Figure 2A). Next, we examined the influence of NORAD on migration and invasion capacities of MM cells. As shown in Figure 2B and C, NORAD knockdown restrained migration by approximately 70% in A375 cells and by nearly 60% in WM35 cells. A similar finding was observed for cell invasion; A375 and WM35 cell invasion was significantly inhibited compared with the normal control group (*P *<* *0.05).

### 3.3 MiR‐205 is a target of NORAD in MM cells

**Figure 3:**
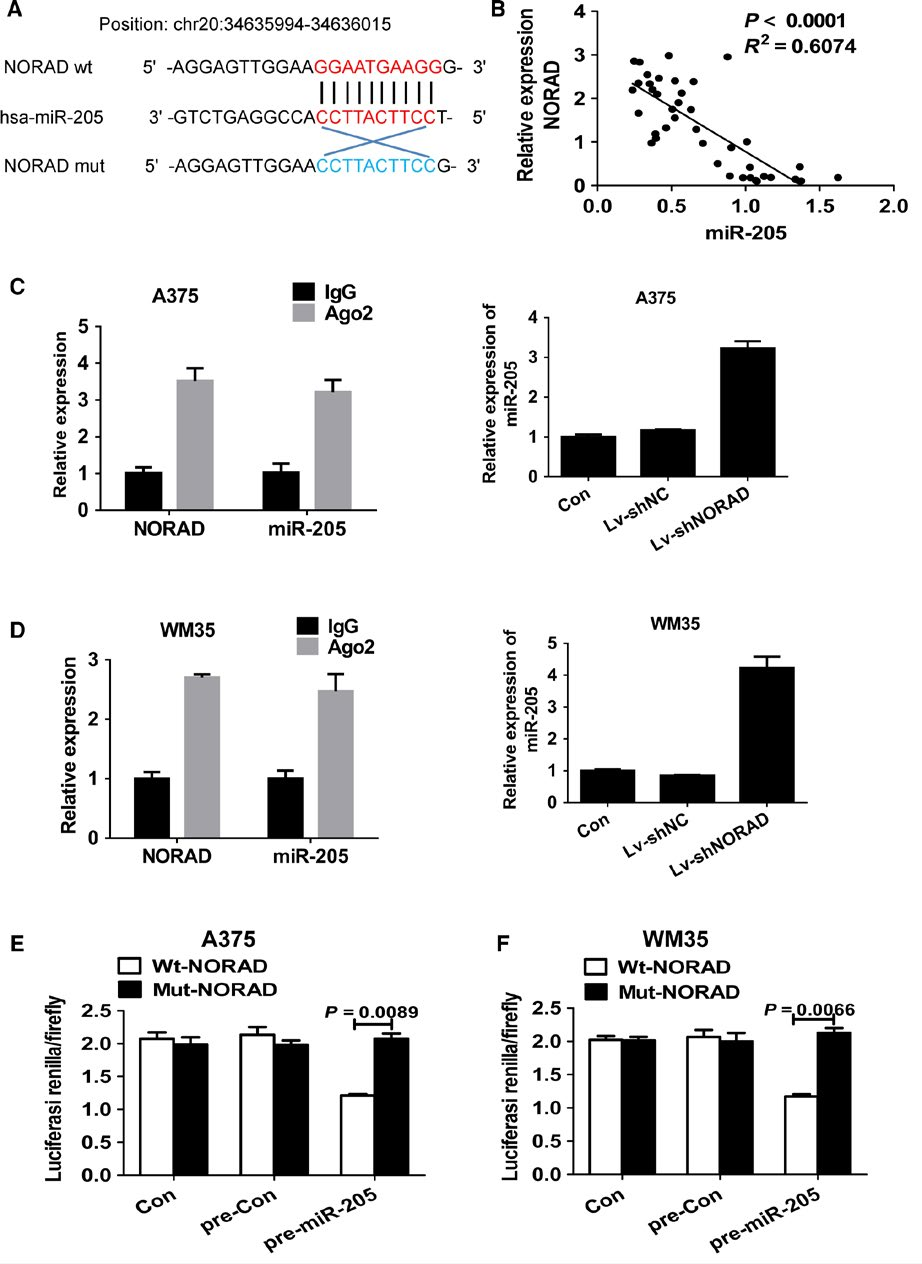
MiR‐205 is a target of NORAD in malignant melanoma cells. (A) Schematic illustration of miR‐205 putative target sites in the transcript of NORAD. (B) Correlation between the expression level of NORAD and that of miR‐205 in 20 matched tissues. Linear regression coefficients and statistical significance is indicated. Association of NORAD and miR‐205 with Ago2 in A357 (C) and WM35 (D) cells. Cellular lysates from A357 and WM35 cells were used for RIP with Ago2 antibody. NORAD and miR‐205 expression levels were detected using qRT‐PCR. The relative luciferase activities were inhibited in the A357 (E) and WM35 (F) cells co‐transfected with wild‐type NORAD vector and pre‐miR‐205, and not with the mutant‐type vector. Firefly luciferase activity was normalized to *Renilla* luciferase

Bioinformatics analysis (StarBase, http://starbase.sysu.edu.cn/browseNcRNA.php) predicted 67 potential targeted miRNAs of NORAD. Of these, miR‐205, miR‐17, miR‐25‐3p, miR‐93‐5p, and miR‐106b‐5p were closely related to melanoma cellular endoplasmic reticulum (ER) stress. The miR‐205 binding site was found in the NORAD transcript (Figure 3A). After detecting the expression levels of the five aforementioned miRNAs in melanoma tissue, only miR‐205 was significantly negatively correlated with NORAD (Figure 3B). An RNA pull‐down experiment was done to determine whether the presence of both miR‐205 and NORAD in RNA‐induced silencing complex (RISC). Both miR‐205 and NORAD were found in the Ago2 pellet in A375 and WM35 cells, knockdown of NORAD elevated the expression of miR‐205 (Figure 3C and D).

To further study if the NORAD is directly targeted miR‐205, the transcript of NORAD was cloned downstream to wild‐type (wt)‐NORAD and a mutant version of NORAD (mut‐NORAD) was also constructed through binding site mutagenesis. The luciferase activity of the cells was decreased with compared to scramble control cells. Furthermore, the putative binding site of the mutant in A375 (Figure 3E) and WM35 (Figure 3F) cells reduced miR‐205‐mediated repression of luciferase activity. The data indicated the binding of miR‐205 to NORAD.

### 3.4 MiR‐205 inhibits MM cell migration and invasion

**Figure 4:**
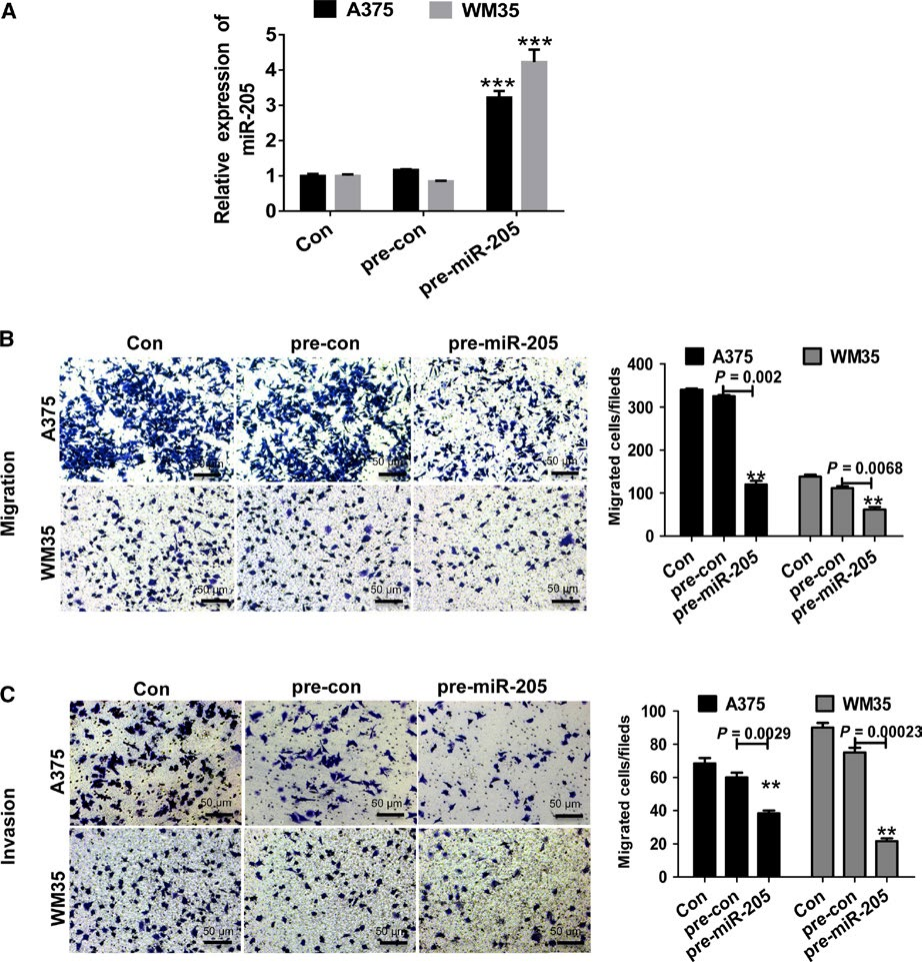
MiR‐205 inhibits migration and invasion of malignant melanoma cells. (A) qPCR was performed to detect the expression of miR‐205 in A357 and WM35 cells after transfection with pre‐miR‐205. (B) The overexpression of miR‐205 inhibited the migration of A357 and WM35 cells. (C) miR‐205 overexpression inhibited the invasion of A357 and WM35 cells. **P ＜ 0.01, ***P <* *0.001 vs normal control (NC). Images were obtained at 100× magnification

To determine if miR‐205 can regulate MM cell migration and invasion, transwell assays were performed through the transfection of precursor of miR‐205 (pre‐miR‐205) or scramble control into A375 and WM35 cells (Figure 4A). Overexpression of miR‐205 induced the inhibition of migration compared to the scramble control (Figure 4B). The corresponding effect on cell invasion was found (Figure 4C) suggesting that miR‐205 can inhibit migration and invasion in MM cells.

### 3.5 Downregulation of NORAD substantially reverses knockdown of miR‐205‐induced migration and invasion

**Figure 5:**
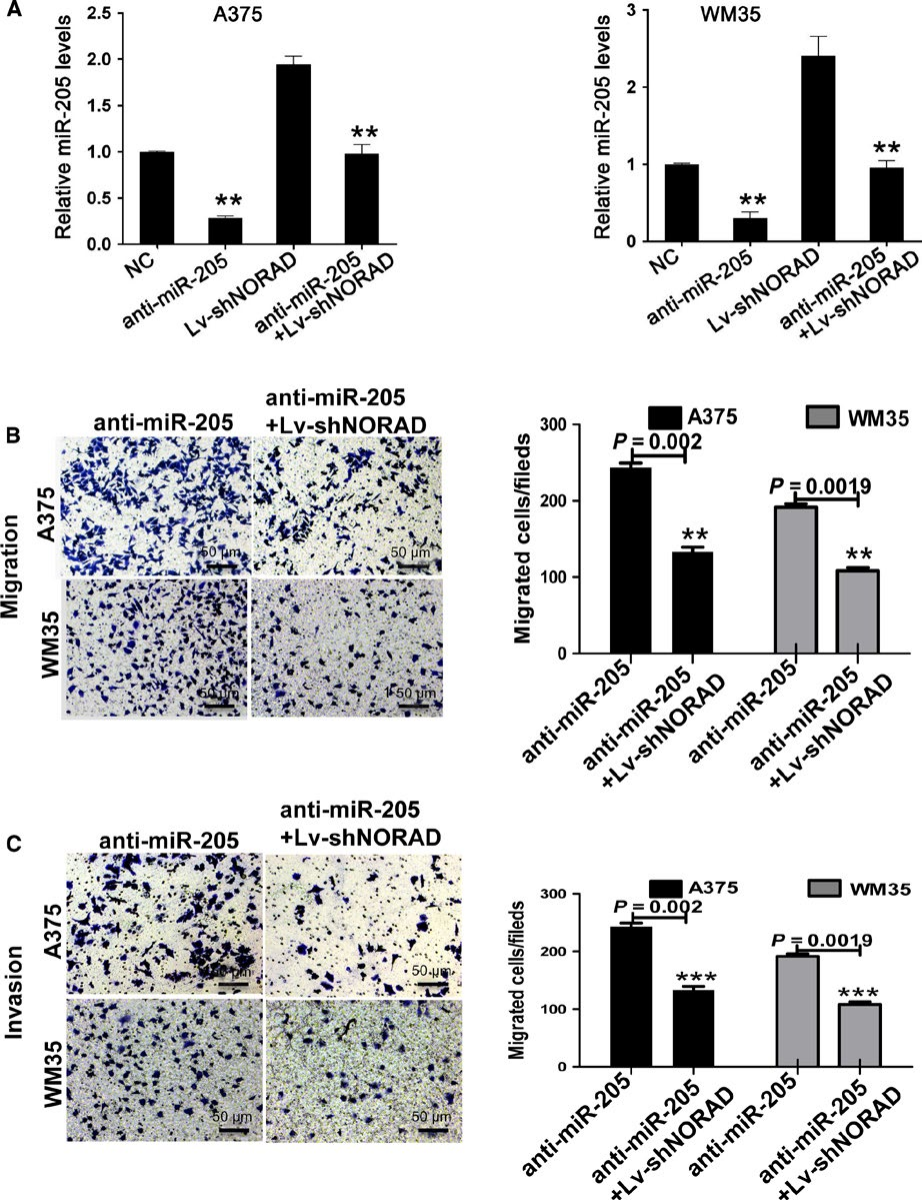
Downregulation of NORAD almost completely reverses knockdown of miR‐205‐induced migration and invasion of malignant melanoma cells. (A) qPCR was performed to detect the expression of miR‐205 in A357 and WM35 cells after transfection with anti‐miR‐205 or Lv‐shNORAD, or with both of them. (B) The downregulation of miR‐205 reversed NORAD knockdown‐mediated inhibition of the migration of A357 and WM35 cells. (C) The downregulation of miR‐205 reversed NORAD knockdown‐mediated inhibition of the invasion of A357 and WM35 cells. **P <* *0.01, ***P < 0.001 vs normal control (NC). Images were obtained at 100× magnification

Based on the observed regulatory effect between NORAD and miR‐205, we further investigated whether NORAD mediated the inhibitory effects of miR‐205 knockdown in tumor migration and invasion. A375 and WM35 cells were transfected with anti‐miR‐205 or shNORAD alone, or co‐transfected with both. The qRT‐PCR results revealed that knockdown of NORAD significantly reversed the downregulation of miR‐205 (Figure 5A). We further evaluated the regulatory effects of downregulated of NORAD, specifically whether the knockdown of miR‐205 affected cancer cell migration and invasion. The downregulation of NORAD largely reversed the inhibitory effect of the deletion of miR‐205 on cell migration and invasion (Figure 5B and C).

### 3.6 Egl nine homolog 2 (EGLN2) is target of miR‐205 and downregulation of NORAD substantially reverses knockdown of miR‐205‐induced ER stress of MM cells

**Figure 6:**
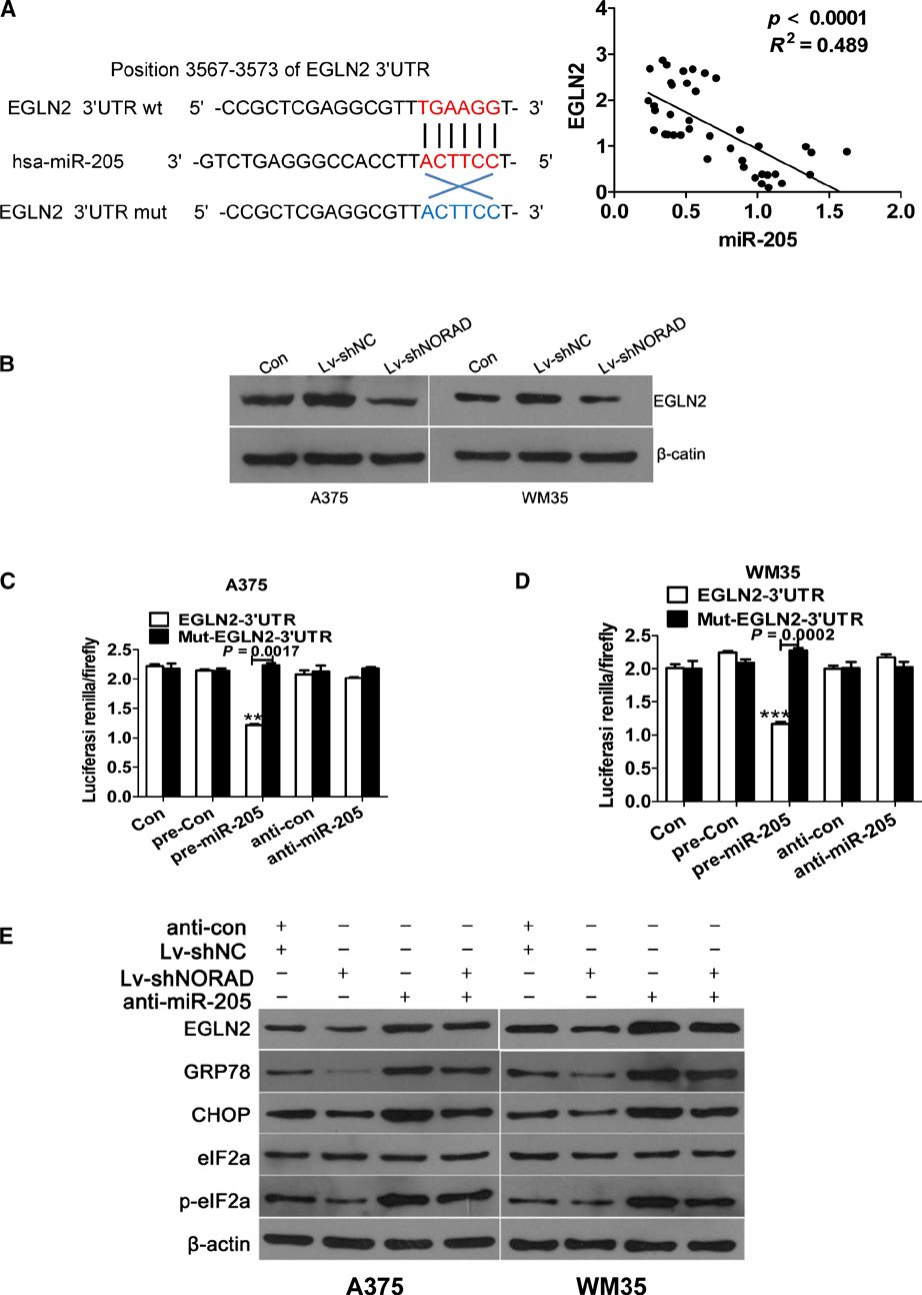
EGLN2 is a direct target of miR‐205 and downregulation of NORAD almost completely reverses knockdown of miR‐205‐induced endopl‐asmic reticulum stress of malignant melanoma cells. (A) Correlation between the expression level of EGLN2 and that of miR‐205 in 20 matched tissues. Linear regression coefficients and statistical significance is indicated. (B) Western blot analysis for EGLN2 after transfection with Lv‐shNORAD. β‐actin was used a loading control. **P < 0.01, ***P <* *0.001 vs normal control (NC) (C, D) The relative luciferase activities were inhibited in the A357 and WM35 cells co‐transfected with wild‐type EGLN2 3′‐UTR vector and pre‐miR‐205, and not with the mutant‐type vector. Firefly luciferase activity was normalized to Renilla luciferase. **P <* *0.01, ***P <* *0.001 vs normal control (NC) (E) Western blot analysis for EGLN2, CHOP, GRP78, eEIF2a, and p‐eEIF2a after treatment. β‐actin was used a loading control

EGLN2 has a binding site for miR‐205. Moreover, the expression of EGLN2 in 40 cases metastasis MM tissues was negatively related with the miR‐205 level (Figure 6A). Interestingly, the stable knockdown of NORAD suppressed the expression of EGLN2 in A375 and WM35 cells (*P *<* *0.05; Figure 6B). In addition, the dual‐luciferase reporter assay revealed that the relative luciferase activity was reduced in A375 and WM35 cells transfected with wild‐type vector after overexpression of miR‐205, while the mut vector reversed this inhibitory effect (Figure 6C and D). The results demonstrated that EGLN2 is a target gene of miR‐205. The regulatory effects of NORAD on miR‐205 knockdown‐induced downregulation of EGLN2 and ER‐related genes were measured by western blot assay. NORAD knockdown dramatically reversed the increased EGLN2 and ER‐related gene levels, including GRP78, CHOP, and eIF2α, which were substantially inhibited by miR‐205(Figure 6E). The results indicated that NORAD can regulate migration, invasion, and ER stress of MM cells by targeting EGLN2 via miR‐205.

### 3.7 Downregulation of NORAD mostly reverses knockdown of miR‐205‐induced tumor growth in nude mice

**Figure 7:**
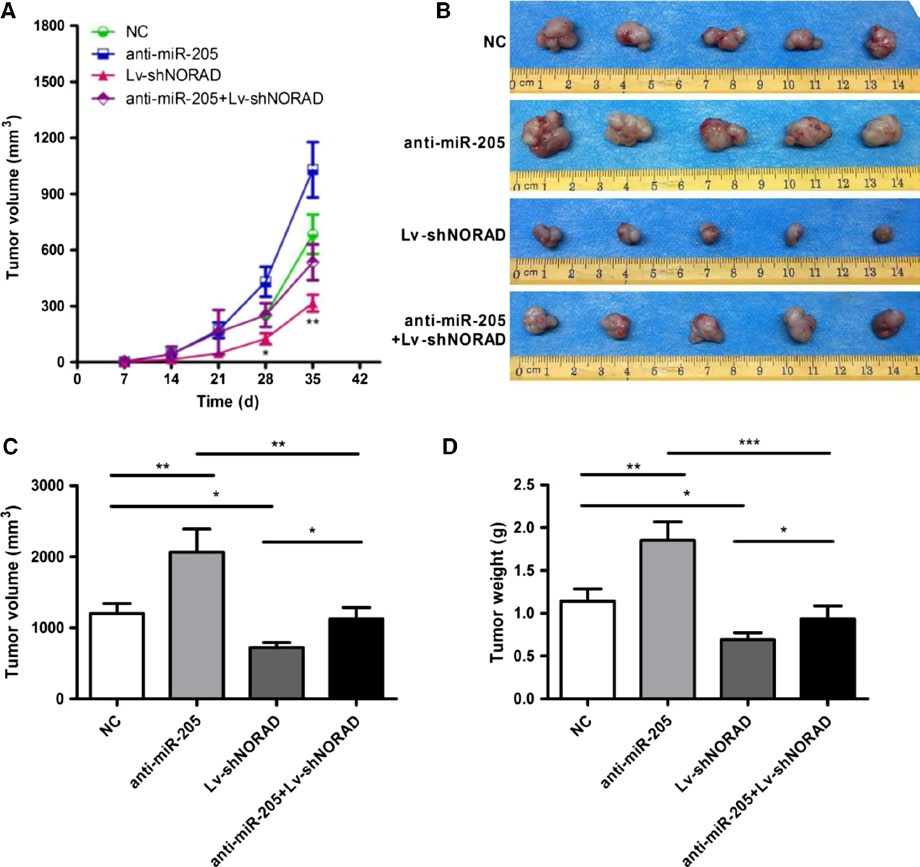
Knockdown of NORAD suppresses tumor growth in nude mice. (A) Growth curve of nude mice with different levels of miR‐33a‐5p expression. Day 0 means the day of injection of tumor cells. (B) Representative images of nude mice and subcutaneous tumors at day 42. Volume (C) and (D) weight of A375 xenografts in each group. *P ＜ 0.05, **P ＜ 0.01, ***P < 0.001 vs normal control (NC)

To investigate the effects of NORAD/miR‐205 axis in vivo, human melanoma A375 cells were subcutaneously inoculated into nude mice. The growth curve of each group (Figure 7A) revealed that the anti‐miR‐205 group of nude mice displayed a higher tumor volume than the negative control, Lv‐shNORAD, and anti‐miR‐205 + Lv‐shNORAD groups (*P* < 0.05). After 35 days, the mice were sacrificed and the tumors were removed (Figure 7B). The mean tumor volume was 2.093 ± 0.353 cm^3^ in the anti‐miR‐205 group, which was higher than the blank vector group, shNORAD, and anti‐miR‐205 + Lv‐shNORAD groups (1.293 ± 0.353, 0.798 ± 0.123, and 1.193 ± 0.233 cm^3^, respectively) (Figure 7C). The anti‐miR‐205 group had higher average tumor weight than that in the blank vector, shNORAD, and anti‐miR‐205 + Lv‐shNORAD groups (Figure 7D). We validated the promoting effects of downregulated miR‐203 on human melanoma xenograft formation, while downregulation of NORAD mostly reversed these effects.

## 4 DISCUSSION

LncRNAs are regulators of various biological processes and they play a critical role in various cancers, including MM. NORAD was identified as a novel lncRNA in response to DNA damage and has a role in the progression of cancer.^13^ However, it is unclear whether NORAD can regulate the migration and invasion of melanoma cells.

We observed high levels of NORAD in melanoma tissues and human malignant melanoma cells, particularly in metastatic MM. NORAD knockdown significantly repressed MM cell migration and invasion. NORAD acts as a molecular sponge in regulating miR‐205. Furthermore, *EGLN2* encodes prolyl hydroxylase 2, which is a key regulator of ER stress and the target of miR‐205. Knockdown of NORAD increased the expression of miR‐205 and subsequently decreased *EGLN2* expression. In addition, knockdown of miR‐205 induced an promotion of migration, invasion, and ER stress in MM cells.

Severe ER stress induced cell death, invasion and alterations of morphology by decreasing the expression of unfolded protein response target genes, including CHOP, GRP78, and EF2a.^14^ ER stress induces epithelial‐mesenchymal transition in lung cancer cells and participates in the progression of metastasis.^15^, ^16^ In addition, ER stress‐induced invasion and migration of breast cancer cells can be suppressed by downregulation of heparanase.^17^ Our data demonstrated that silencing of NORAD can inhibit ER stress. Although the present study did not provide direct evidence whether changes in ER stress were required for the promotion of invasion and migration of MM cells caused by NORAD, further investigating the role of NORAD and ER stress in cell invasion and migration may provide an novel thought to MM therapies.

The “competing endogenous RNAs” theory posits a novel mechanism of between lncRNA and miRNA.^18^ We found that miR‐205 may be a target of NORAD by bioinformatic prediction based on complementary sequences. RIP and luciferase reporter assay analyses demonstrated that miR‐205 has direct binding sites on NORAD. The function of miR‐205 as a tumor suppressor was firstly found in breast cancer,^19^ and then on, downregulated of miR‐205 and the tumor suppressor role of in the occurrence and development of several types of cancer such as cervical cancer,^20^ colorectal cancer,^21^ and bladder cancer^22^ has been established. However, MiRNAs can target various genes and single gene can be modulated by many MiRNAs, therefore, the biological outcomes of miRNA activation may change with cell content and stimulus, which provides potential explanations for the complicated and controversial functions of miRNA. MiR‐205 is a “double‐edged sword” in cancer, some studies described miR‐205 is also upregulated in some cancers, such as ovarian cancer and head and neck cancers.^23^, ^24^According to our observations and other studies, we confirmed that miR‐205 is downregulated in MM.^25^, ^26^, ^27^ Another study also described the downregulation of miR‐205 in human MM tissues and cells,^28^ which heralds a poor prognosis for melanoma patients.^29^ Upregulation of MiR‐205 also restrained growth, migration, and invasion of MM cells through the targeting of erbb3, E2F1, and the zinc‐finger E‐box binding homeobox2.^28^, ^30^, ^31^, ^32^, ^33^ These observations are identical to our observations that the up‐regulation of miR‐205 can inhibit MM cell migration and invasion, and that EGLN2 is a target gene of miR‐205.

EGLN2 is believed to be the major regulator of the hypoxia response, participating in diverse biological processes including cell invasion and cell death.^34^ *EGLN2* is an invasion‐associated gene. It is abundantly expressed in highly invasive lung cancer cells and has been recognized as a prognostic factor for non‐small cell lung cancer. Moreover, miR‐205 could bind to the EGLN2 3′‐UTR and dampen transcription level of EGLN2, which regulates the level of intracellular reactive oxygen species, cell invasion, and ER stress.^35^

In our study, stable knockdown of NORAD dampened expression of EGLN2 in A375 and WM35 cells, and the regulatory effects of NORAD on the expression of EGLN2 and ER‐related genes, for example,GRP78, CHOP, and eIF2α were largely reversed by miR‐205. WM35 cells are poorly tumorigenic and can even be non‐tumorigenic in nude mice. Thus, only A375 cells were selected to further confirm the effect of the NORAD/miR‐205 axis. In the experiment involving nude mice, the downregulation of miR‐205 promoted growth of melanoma xenografts, while the knockdown of NORAD almost completely reversed the promotion of growth. Thus, it is reasonable to conclude that the NORAD/miR‐205 axis inhibits the migration and invasion of MM cells, and it may caused by the induction of ER stress.

In conclusion, the overexpression of NORAD is associated with metastasis of MM cells. NORAD interacts with miR‐205 and acts as an oncogene, which enhances the malignancy of MM cells. The present data reveal the participation of NORAD in the tumorigenesis of MM cells, and may indicate a novel therapeutic target.

## CONFLICT OF INTEREST

The authors declare no conflict of interest.
